# An Assessment of Implant Stability and Bite Force Following Early and Delayed Dental Implant Placement

**DOI:** 10.7759/cureus.112309

**Published:** 2026-07-08

**Authors:** Jay Gohil, Shveta Jain, Sreesha S Sreedhar, Syed Ashfaq Ur Rahman, Shefali Sharma, Prem Sagar Yekula

**Affiliations:** 1 Department of Prosthodontics, K. M. Shah Dental College and Hospital, Sumandeep Vidyapeeth (Deemed to be University), Vadodara, IND; 2 Department of Prosthodontics, Jain Dental and Implant Care Centre, Dehradun, IND; 3 Department of Oral and Maxillofacial Surgery, Azeezia College of Dental Sciences and Research, Kollam, IND; 4 Department of Oral and Maxillofacial Surgery, Kamineni Institute of Dental Sciences, Hyderabad, IND; 5 Department of Periodontics, Uttaranchal Dental and Medical Research Institute, Dehradun, IND; 6 Department of Prosthodontics, Sibar Institute of Dental Sciences, Guntur, IND

**Keywords:** bite force, dental implants, implant stability, osseointegration, tooth extraction

## Abstract

Introduction

The timing of dental implant placement following tooth extraction is an important clinical consideration because it may influence osseointegration and functional rehabilitation. Although both early and delayed implant placement protocols are widely used, evidence regarding their effects on implant stability and masticatory function is limited. This study aimed to compare early and delayed dental implant placements in terms of implant stability and bite force.

Materials and methods

This prospective observational study was conducted at the Department of Prosthodontics, K. M. Shah Dental College and Hospital, Sumandeep Vidyapeeth (Deemed to be University), Vadodara, Gujarat, India. Sixty patients were categorized into two groups of 30 participants in each group. The early implant group received implants four to eight weeks after tooth extraction, whereas the delayed implant group underwent implant placement after a minimum healing period of six months. Implant stability was assessed using resonance frequency analysis (RFA) and expressed as implant stability quotient (ISQ) values. Functional rehabilitation was evaluated by measuring the bite force using a digital occlusal force analyzer. Assessments were performed at baseline and six months after prosthetic loading. Statistical analyses were conducted using paired and independent t-tests, with statistical significance set at p < 0.05.

Results

Both groups demonstrated significant improvements in implant stability and bite force over the six-month follow-up period (p = 0.001). In the early implant group, the mean ISQ values increased from 62.4 ± 4.8 to 74.6 ± 3.9, whereas the delayed implant group showed an increase from 58.7 ± 5.2 to 71.2 ± 4.6. The bite force increased from 128.4 ± 22.6 N to 218.3 ± 31.4 N in the early implant group and from 134.7 ± 25.1 N to 204.6 ± 28.9 N in the delayed implant group. The early implant group demonstrated significantly higher implant stability at baseline and six months (p < 0.05). Although the bite force values at individual time points were comparable between the groups, the increase in bite force over time was significantly greater in the early implant group (p = 0.006).

Conclusions

Both early and delayed implant placement protocols achieved successful osseointegration and functional rehabilitation in the present study. However, early implant placement demonstrated superior implant stability and greater improvement in bite force, suggesting that it may provide favorable clinical outcomes when appropriate case selection and treatment planning are performed.

## Introduction

Replacing missing teeth with endosseous dental implants has become a predictable and widely accepted treatment option in contemporary dentistry. High long-term survival rates and favorable patient-reported outcomes have established implant therapy as an integral component of oral rehabilitation. Nevertheless, the timing of implant placement following tooth extraction continues to be an important clinical consideration because it may influence both the healing process and the subsequent functional outcomes [1,2].

Following tooth extraction, the alveolar ridge undergoes a series of dimensional and structural changes, characterized by bone remodeling and soft tissue adaptation. These post-extraction alterations may affect implant positioning, primary stability, and prosthetic rehabilitation outcomes. Traditionally, implant placement has been delayed until complete healing of the extraction socket occurs. However, the increasing emphasis on reducing treatment duration and preserving alveolar architecture has encouraged the adoption of earlier implant placement protocols [3].

A fundamental determinant of implant success is the achievement and maintenance of implant stability (IS). Implant stability reflects the mechanical engagement between the implant surface and the surrounding bone and plays a crucial role in successful osseointegration. Resonance frequency analysis (RFA) is widely used for the objective assessment of implant stability because it provides reliable and reproducible implant stability quotient (ISQ) values throughout the healing period [4]. Therefore, the evaluation of implant stability offers valuable insights into the biological and mechanical behaviors of implants placed under different clinical conditions.

In addition to successful osseointegration, restoration of oral function is the primary objective of implant-supported rehabilitation. Masticatory performance improves considerably following the replacement of missing teeth, and bite force is an objective indicator of functional recovery. The measurement of bite force allows a quantitative assessment of the patient's ability to generate occlusal loading and provides information regarding adaptation to implant-supported prostheses [5]. Consequently, the combined assessment of implant stability and bite force can provide a comprehensive understanding of both biological integration and functional rehabilitation of the implant.

Although numerous studies have evaluated implant survival rates associated with different placement protocols, fewer studies have simultaneously examined mechanical stability and functional performance following early and delayed implant placements. Understanding the relationship between implant placement timing, osseointegration, and masticatory function may assist clinicians in selecting the most appropriate treatment approach while balancing the treatment duration and clinical outcomes [6-8].

Therefore, the present study was undertaken to compare early and delayed dental implant placement protocols with respect to implant stability and functional rehabilitation. Implant stability was assessed using RFA, whereas functional recovery was evaluated through bite force measurements. The study aimed to determine changes in these parameters over time and to compare the clinical outcomes associated with the two implant placement approaches. It was hypothesized that early implant placement would be associated with higher implant stability and greater improvement in bite force than delayed implant placement. Implant stability and bite force were considered co-primary outcome measures.

## Materials and methods

This prospective observational comparative study was conducted in the Department of Prosthodontics, K. M. Shah Dental College and Hospital, Sumandeep Vidyapeeth (Deemed to be University), Vadodara, Gujarat, India. Ethical approval was obtained from the Institutional Ethics Committee of the college (SVIEC/Dent/2023/april/34; approval dated 12 April 2023). Written informed consent was obtained from all participants before enrolment. Patient confidentiality was maintained throughout the study, and the participants were informed of their right to withdraw at any stage without affecting their treatment. The study was conducted over 12 months, from April 2023 to April 2024, and included patient recruitment, implant placement, prosthetic rehabilitation, and follow-up assessments. Outcome measurements were recorded at baseline, before prosthetic loading, and six months after loading.

A total of 60 patients requiring implant-supported rehabilitation were included in the study and were categorized into two groups of 30 participants each according to the timing of implant placement following tooth extraction. The early implant group comprised patients who underwent implant placement four to eight weeks after extraction during the early healing phase, whereas the delayed implant group included patients who underwent implant placement after a minimum healing period of six months. Baseline demographic and implant-related characteristics were assessed to ensure comparability between groups.

An a priori sample size calculation was performed using G*Power (version 3.1.9.7; Heinrich Heine University, Düsseldorf, Germany) based on an independent t-test to compare the means between two independent groups. Based on previously published data reporting a mean ISQ of 62.1 ± 5.3 for early implants and 57.8 ± 5.6 for delayed implants, with an anticipated mean difference of 4.3 ISQ units and a pooled standard deviation (SD) of approximately 5.0, the following parameters were applied: a two-tailed significance level of α = 0.05 and statistical power of 80%. This yielded a minimum required sample size of 27 participants per group for the study. Accounting for an anticipated attrition and dropout rate of 10%, the final adjusted sample size was set at 30 participants per group, resulting in a total sample size of 60 participants.

Patients aged 18-50 years who required extraction of at least one non-restorable tooth in the maxilla or mandible were considered eligible for inclusion. Adequate bone volume to accommodate implants of at least 3.5 mm in diameter and 10 mm in length without the need for augmentation procedures was required. Patients with sufficient interarch space and appropriate opposing dentition for prosthetic rehabilitation were included in the study. Only systemically healthy individuals or those with well-controlled systemic diseases were enrolled. Both non-smokers and light smokers were eligible for participation. Patients with uncontrolled systemic diseases, active periodontal disease, previous head and neck radiotherapy, parafunctional habits, pregnancy, or the need for adjunctive surgical procedures, such as bone grafting or sinus augmentation, were excluded.

Implant installation protocols have been classified into different categories based on the timing of implant placement following tooth extraction and the stage of socket healing. Hämmerle et al. [9] first proposed a classification system that was subsequently refined by Buser et al. [10], categorizing implant placement into four clinical protocols: type I (immediate implant placement), performed immediately after tooth extraction; type II (early implant placement with soft tissue healing), performed four to eight weeks after extraction; type III (early implant placement with partial bone healing), performed 12-16 weeks after extraction; and type IV (delayed implant placement), performed after complete soft and hard tissue healing, generally beyond four months following extraction. This study evaluated type II and type IV implant placement protocols, referred to as the early and delayed implant groups, respectively.

All implant surgeries were performed under aseptic conditions, using a standardized surgical protocol. Local anesthesia was achieved using 2% lignocaine with 1:200,000 adrenaline (Lignox 2% A; Indoco Remedies Ltd., Mumbai, India). Osteotomy preparation was performed under copious saline irrigation according to the manufacturer's recommendations. Endosseous titanium dental implants (Adin Dental Implant Systems Ltd., Afula, Israel) with a minimum diameter of 3.5 mm and a length of 10 mm were placed. Adequate primary stability was achieved at the time of implant placement in all cases. Healing abutments were placed in implants that achieved adequate primary stability and were managed using a transmucosal healing protocol, whereas implants requiring submerged healing received cover screws. All implants underwent conventional prosthetic loading after a healing period of three months. Prosthetic rehabilitation was initiated only after satisfactory clinical implant stability had been confirmed. All implants were placed by one implantologist with more than five years of experience.

Implant stability was evaluated using RFA with an Osstell ISQ device (Osstell AB, Gothenburg, Sweden). A compatible SmartPeg was attached to each implant, and measurements were recorded in both the buccolingual and mesiodistal directions. The mean ISQ value obtained from the measurements was used for the analysis. Assessments were performed at baseline and six months after prosthetic loading. Functional rehabilitation was assessed by measuring the bite force using a digital occlusal force analyzer (Bite Force Meter, Nagano Keiki Co. Ltd., Tokyo, Japan). The participants were instructed to bite maximally on the sensor positioned at the implant-supported prosthetic site. Three consecutive readings were recorded for each participant, and the average value, expressed in Newtons (N), was used for statistical analysis.

Statistical analyses were performed using SPSS Statistics for Windows, version 26.0 (IBM Corp., Armonk, NY). Continuous variables were expressed as mean ± SD, whereas categorical variables were presented as frequencies and percentages. The normality of the data distribution was assessed using the Shapiro-Wilk test. Intragroup comparisons between baseline and six-month measurements were performed using the paired t-test, while intergroup comparisons were analyzed using the independent t-test. Categorical variables were evaluated using the chi-square test. Statistical significance was set at p < 0.05.

## Results

Table 1 presents the demographic and implant-related characteristics of the participants. The mean age was 34.60 ± 8.20 years in the early implant group and 38.10 ± 9.40 years in the delayed implant group (p = 0.130). No statistically significant differences were observed between the groups with respect to sex distribution, implant location, prosthesis type, implant diameter, implant length, or bone quality (all p > 0.05), indicating baseline comparability and reducing the likelihood of confounding effects.

**Table 1:** Demographic and implant characteristics of the study participants (N = 60) An independent t-test was used for continuous variables; a chi-square test was used for categorical variables; p < 0.05 was considered statistically significant df: degrees of freedom; SD: standard deviation; χ²: chi-square test; t: independent t-test

Characteristic		Early implant group (n = 30)	Delayed implant group (n = 30)	Test statistics (df)	P-value
Age (years)	Mean ± SD	34.60 ± 8.20	38.10 ± 9.40	t = 1.54 (58)	0.130
	Range	22 – 54	24 – 58		
Sex, n (%)	Male	18 (60.0)	17 (56.7)	χ² = 0.07 (1)	0.793
	Female	12 (40.0)	13 (43.3)		
Implant location, n (%)	Anterior	12 (40.0)	14 (46.7)	χ² = 0.27 (1)	0.602
	Posterior	18 (60.0)	16 (53.3)		
Prosthesis type, n (%)	Single crown	21 (70.0)	19 (63.3)	χ² = 0.30 (1)	0.584
	Fixed partial denture	9 (30.0)	11 (36.7)		
Implant diameter (mm)	Mean ± SD	3.90 ± 0.40	4.00 ± 0.30	t = 1.10 (58)	0.278
Implant length (mm)	Mean ± SD	10.80 ± 1.40	11.10 ± 1.30	t = 0.86 (58)	0.393
Bone quality, n (%)	Type II	13 (43.3)	15 (50.0)	χ² = 0.27 (1)	0.605
	Type III	17 (56.7)	15 (50.0)		

Table 2 shows the intragroup comparison of implant stability and bite force between baseline and six months. In the early implant group, mean ISQ values increased significantly from 62.40 ± 4.80 at baseline to 74.60 ± 3.90 at six months (p = 0.001), while bite force increased from 128.40 ± 22.60 N to 218.30 ± 31.40 N (p = 0.001). Similarly, in the delayed implant group, the mean ISQ values increased significantly from 58.70 ± 5.20 to 71.20 ± 4.60 (p = 0.001), and bite force improved from 134.70 ± 25.10 N to 204.60 ± 28.90 N (p = 0.001). These findings demonstrated successful osseointegration and functional recovery in both groups during the six-month follow-up period.

**Table 2:** Intragroup comparison of implant stability and bite force between baseline and six months within early and delayed implant groups ^*^P < 0.05 considered statistically significant; t: paired t-test was used for within-group comparison SD: standard deviation; df: degrees of freedom; ISQ: implant stability quotient

Groups	Outcome variables	Baseline (mean ± SD)	Sixth month (mean ± SD)	Difference (mean ± SD)	Paired t statistic (df)	P-value
Early implant group (n = 30)	Resonance frequency analysis, ISQ values	62.40 ± 4.80	74.60 ± 3.90	+12.20 ± 3.10	17.60 (29)	0.001^*^
	Bite force measurements (newtons)	128.40 ± 22.60	218.30 ± 31.40	+89.90 ± 18.40	21.85 (29)	0.001^*^
Delayed implant group (n = 30)	Resonance frequency analysis, ISQ values	58.70 ± 5.20	71.20 ± 4.60	+12.50 ± 3.60	15.53 (29)	0.001^*^
	Bite force measurements (newtons)	134.70 ± 25.10	204.60 ± 28.90	+69.90 ± 16.20	19.20 (29)	0.001^*^

Table 3 presents the intergroup comparison of implant stability and bite force. The early implant group exhibited significantly higher ISQ values than the delayed implant group at baseline (p = 0.022) and six months after loading (p = 0.019). Although no significant difference was observed in the magnitude of ISQ improvement between the groups (p = 0.731), the early implant group maintained a higher stability throughout the study period. Bite force values were comparable between the groups at baseline (p = 0.557) and six months (p = 0.230). However, the increase in bite force from baseline to six months was significantly greater in the early implant group than in the delayed implant group (p = 0.006). Overall, early implant placement was associated with superior implant stability and greater functional improvement compared to delayed implant placement.

**Table 3:** Intergroup comparison of implant stability and bite force between early and delayed implant placement at baseline and six months ^*^P < 0.05 considered statistically significant; t: independent t-test was used for between-group comparison SD: standard deviation; CI: confidence interval; df: degrees of freedom; ISQ: implant stability quotient

Outcome	Time point	Early implant group (mean ± SD)	Delayed implant group (mean ± SD)	Mean difference (early – delayed)	95% CI	Independent t statistic (df)	P-value
Resonance frequency analysis, ISQ values	Baseline	62.40 ± 4.80	58.70 ± 5.20	+3.70	0.6 to 6.8	2.39 (58)	0.022^*^
	6 months (post‑loading)	74.60 ± 3.90	71.20 ± 4.60	+3.40	0.6 to 6.0	2.45 (58)	0.019^*^
	Change (Δ baseline → 6 months)	+12.20 ± 3.10	+12.50 ± 3.60	–0.30	–2.04 to 1.44	-0.34 (58)	0.731
Bite force measurements (newtons)	Baseline	128.40 ± 22.60	134.70 ± 25.10	–6.30	–19.4 to 10.6	0.59 (58)	0.557
	6 months (post‑loading)	218.30 ± 31.40	204.60 ± 28.90	+13.70	–7.6 to 30.6	1.22 (58)	0.230
	Change (Δ baseline → 6 months)	+89.90 ± 18.40	+69.90 ± 16.20	+20.00	8.0 to 28.8	2.95 (58)	0.006*

## Discussion

This prospective comparative study investigated the effect of implant placement timing on implant stability and functional rehabilitation by comparing early and delayed implant placement protocols. The findings demonstrated that both treatment approaches resulted in significant improvements in implant stability and bite force over the six-month follow-up period, indicating successful osseointegration and restoration of masticatory function. However, implants placed during the early healing phase exhibited significantly higher stability values and greater improvement in bite force than implants placed after complete socket healing.

Implant stability is a critical determinant of long-term implant success because it reflects the quality of the bone-implant interface and the progression of the osseointegration process. In the present study, the ISQ values increased significantly in both groups from baseline to six months, confirming the progressive biological integration of the implants within the surrounding bone [11]. The higher ISQ values observed in the early implant group at both assessment periods may be attributed to the preservation of alveolar ridge dimensions during the early stages of socket healing.

Following tooth extraction, substantial dimensional changes occur within the alveolar ridge, particularly during the first few months, owing to bone remodeling and maturation processes [12,13]. Placement of implants during the early healing phase may utilize the existing socket architecture before extensive ridge resorption occurs, thereby promoting favorable primary stability and subsequent osseointegration. These findings suggest that preserving the healing socket environment may contribute to improved implant stability outcomes. Similar observations were reported by Bischof et al. [14], who demonstrated progressive increases in implant stability during healing and emphasized the influence of implant placement time on stability patterns during osseointegration.

The results also demonstrated a substantial improvement in bite force in both groups following prosthetic loading. The recovery of bite force reflects the functional adaptation of the stomatognathic system and the successful transfer of occlusal loads through implant-supported restorations. The significantly greater increase in bite force observed in the early implant group suggests enhanced functional rehabilitation and more efficient biomechanical integration. Earlier restoration of the edentulous area may contribute to improved neuromuscular adaptation and occlusal performance in patients. Fontijn-Tekamp et al. [5] reported that implant-supported prostheses significantly improve chewing efficiency and biting performance, highlighting the role of implant therapy in restoring functional capacity.

A recent cohort study by Mohammed et al. [15] reported a significant association between bite force and implant stability, demonstrating that improvements in the ISQ values were accompanied by corresponding increases in bite force over time. The authors suggested that the relationship between these variables evolves dynamically during osseointegration and is influenced by factors such as implant location, patient characteristics, and the duration of functional loading. The greater improvement in bite force observed in the present study may therefore reflect the favorable stability characteristics achieved in the early implant group, rather than a direct effect of implant placement timing alone.

The findings of the present study are further supported by systematic evidence evaluating different implant-placement protocols. Patel et al. [7] reported that immediate and delayed implant placement protocols demonstrate comparable survival rates when appropriate case selection and surgical principles are followed. Nevertheless, earlier placement approaches may provide advantages related to treatment duration and patient-centered outcomes. These observations correspond with the superior implant stability and functional improvement identified in the present study.

Although the magnitude of improvement in ISQ values was comparable between the groups, implants placed during the early healing phase maintained a significantly higher stability throughout the observation period. This finding suggests that both protocols can achieve successful osseointegration, but earlier placement may offer a more favorable biological environment during healing. The present findings are also consistent with the systematic review and meta-analysis conducted by Garcia-Sanchez et al. [8], who reported comparable implant survival among immediate, early, and delayed placement protocols, while emphasizing the importance of careful patient selection and surgical execution. Similarly, Block et al. [16] observed favorable clinical outcomes and progressive improvements in implant function, irrespective of placement timing, supporting the concept that successful rehabilitation depends on achieving adequate stability and functional loading.

An important strength of the present study is the use of objective outcome measures to evaluate both the biological and functional aspects of implant therapy. RFA provides a reproducible assessment of implant stability, whereas bite force measurement offers quantitative information regarding the restoration of masticatory performance. Furthermore, the baseline demographic and implant-related characteristics were comparable between the groups, reducing the influence of potential confounding factors and enhancing the validity of the findings. The early implant group demonstrated higher implant stability throughout the study period. However, because baseline ISQ values were also significantly higher in this group, these findings should be interpreted as an association rather than evidence of a causal effect of implant placement timing.

The clinical implications of the present study suggest that early implant placement may be considered a predictable treatment approach capable of providing superior implant stability and enhanced functional recovery while simultaneously reducing overall treatment duration. Preservation of the alveolar bone architecture and early prosthetic rehabilitation may contribute to improved patient satisfaction and treatment efficiency. Nevertheless, successful outcomes remain dependent on appropriate patient selection, adequate bone volume, the absence of active infection, and adherence to meticulous surgical protocols.

Despite these encouraging findings, there are certain limitations to this study. The follow-up period was limited to six months; therefore, it does not provide information regarding long-term implant survival and maintenance. The observational nature of this study may have introduced selection bias. Additionally, variables such as implant surface characteristics, occlusal loading patterns, bone density variations, and patient-specific healing responses were not investigated in detail. Future longitudinal studies with larger sample sizes and randomized controlled designs are recommended to further clarify the influence of implant placement timing on long-term clinical and functional outcomes.

## Conclusions

Within the limitations of this prospective observational study, both early and delayed implant placement protocols were associated with significant improvements in implant stability and functional performance over the six-month follow-up period. Successful osseointegration and functional rehabilitation were achieved in both groups. The early implant placement group showed higher implant stability and a greater increase in bite force compared with the delayed implant placement group. However, because baseline implant stability differed significantly between the groups, these findings should be interpreted as an association rather than as evidence of a causal effect of implant placement timing. Early implant placement may represent a favorable treatment approach in appropriately selected patients, provided adequate bone volume, careful case selection, and sound surgical and prosthetic principles are maintained. Further long-term prospective studies, particularly randomized controlled trials, are warranted to confirm these findings and to clarify the influence of implant placement timing on implant stability, survival, and functional outcomes.
